# Identification of the pyroptosis-related gene signature and risk score model for esophageal squamous cell carcinoma

**DOI:** 10.18632/aging.204661

**Published:** 2023-04-17

**Authors:** Minghong Pan, Yuanyong Wang, Zhaoyang Wang, Changjian Shao, Yingtong Feng, Peng Ding, Hongtao Duan, Xiaoya Ren, Weixun Duan, Zhiqiang Ma, Xiaolong Yan

**Affiliations:** 1 Department of Thoracic Surgery, Tangdu Hospital, The Air Force Military Medical University, Xi’an 710038, China; 2 Department of Cardiothoracic Surgery, The Affiliated Huaihai Hospital of Xuzhou Medical University/The 71st Group Army Hospital of PLA, Xuzhou 221004, China; 3 Department of Cardiovascular Surgery, Xijing Hospital, The Air Force Military Medical University, Xi’an 710038, China; 4 Department of Medical Oncology, Senior Department of Oncology, Chinese PLA General Hospital, The Fifth Medical Center, Beijing 100853, China

**Keywords:** pyroptosis, ESCC, risk score, tumor microenvironment

## Abstract

Advanced esophageal squamous cell carcinoma (ESCC) still has a dismal prognostic outcome. However, the current approaches are unable to evaluate patient survival. Pyroptosis represents a novel programmed cell death type which widely investigated in various disorders and can influence tumor growth, migration, and invasion. Furthermore, few existing studies have used pyroptosis-related genes (PRGs) to construct a model for predicting ESCC survival. Therefore, the present study utilized bioinformatics approaches for analyzing ESCC patient data obtained from the TCGA database to construct the prognostic risk model and applied it to the GSE53625 dataset for validation. There were 12 differentially expressed PRGs in healthy and ESCC tissue samples, among which eight were selected through univariate and LASSO cox regression for constructing the prognostic risk model. According to K-M and ROC curve analyses, our eight-gene model might be useful in predicting ESCC prognostic outcomes. Based on the cell validation analysis, C2, CD14, RTP4, FCER3A, and SLC7A7 were expressed higher in KYSE410 and KYSE510 than in normal cells (HET-1A). Hence, ESCC patient prognostic outcomes can be assessed by our PRGs-based risk model. Further, these PRGs may also serve as therapeutic targets.

## INTRODUCTION

Esophageal cancer (EC) ranks 6th among all factors inducing cancer-associated mortality worldwide and exhibits a growing incidence. EC is divided into esophageal adenocarcinoma and esophageal squamous cell carcinoma (ESCC). In Asia, ESCC represents a predominant histological subtype [1]. ESCC patients still have a dismal prognosis despite great progress in treatments. Moreover, some ESCC cases develop resistance to targeted therapies, immunotherapy, and chemotherapy, leading to cancer recurrence and mortality [2, 3]. Such cases of treatment failure may be caused by ESCC heterogeneity [4, 5]. Therefore, identifying ESCC subtypes is of great significance for predicting patient prognostic outcomes and developing individualized treatments.

Pyroptosis is a form of gasdermin-mediated programmed cell death that causes persistent swelling of cells when the cytomembrane ruptures, leading to cellular content leakage [6, 7]. Cancer cells can generate massive antigens during pyroptosis that induce systemic immunity and inhibit cancer growth [8]. Moreover, Gasdermin E (GSDME), a crucial pyroptosis-related protein, induces tumor adaptive immunity by promoting macrophage-mediated phagocytosis, thereby adding to the difficulty of immune evasion of cancer cells [9, 10]. Immune cells infiltrate more readily into GSDME over-expressing tumors compared to GSDME-deficient ones [7, 9]. Therefore, pyroptosis is a significant direction of antitumor therapy. However, there are few studies on the relationship of ESCC prognosis with pyroptosis-related gene (PRG) levels [11], and no further study has been conducted to stratify ESCC into different molecular subtypes to explore the relationship with immune response.

With the development of high-throughput sequencing (HTS), we have gained a comprehensive understanding of tumor gene expression patterns, and ESCC cases show great heterogeneity in the prognosis of combined immunotherapy, and it is greatly significant to provide personalized therapy for subtype classification of patients with ESCC [12, 13]. The present study conducted preliminary research to identify ESCC-related PRGs subtypes based on HTS data. This study investigated the functions of pyroptosis in diverse subtypes, which is beneficial for diagnosis, prognosis, and individualized treatment for ESCC cases.

## MATERIALS AND METHODS

### Datasets and patients

We obtained mRNA expression profiles (Workflow Type: HT seq-FPKM) and matched clinical data of ESCC cases at the Cancer Genome Atlas (TCGA) website (https://portal.gdc.cancer.gov/repository) as a training cohort. Simultaneously, 99 ESCC case samples (including 11 healthy and 88 ESCC tissue samples) were also obtained. The GSE53625 dataset of Gene Expression Omnibus (GEO) (http://www.ncbi.nlm.nih.gov/geo/) was adopted to be a validation cohort. There were 275 ESCC cases enrolled altogether, excluding adenocarcinoma, and their details are in Table 1. This work adopted Affymetrix Human Genome U133 Plus 2.0 Array platform to study GEO samples. Clinicopathological characteristics (age, sex, grade, TNM stage, tobacco, alcohol) of cases were also extracted. Samples without detailed clinical or prognostic information were eliminated from these two databases.

**Table 1:** Clinical information of ESCC patients from TCGA and GEO.

		**TCGA**	**GEO**
		***n* = 96**	***n* = 179**
Age	≥60	39	90
	<60	57	89
Gender	Female	15	33
	Male	81	146
T	T1~2	41	39
	T3~4	55	140
N	N0	52	83
	N1~2, x	44	96
M	M0	86	179
	M1, x	10	0
Stage	Stage I	7	10
	Stage II	57	77
	Stage III	27	92
	Stage IV	5	0

### DEGs identification

We obtained 27 PRGs in Gene Set Enrichment Analysis (GSEA) (https://www.gsea-msigdb.org/gsea). Their expression patterns were obtained from the TCGA database. Moreover, DEGs were identified using the R software “limma” package with a *P* < 0.05 threshold. Furthermore, potential gene-gene interactions were searched, and the DEGs-based protein-protein interaction (PPI) network was built based on the STRING database (http://string-db.org/).

### Risk score model establishment based on univariate Cox as well as LASSO Cox regression

This work conducted univariate regression to screen the prognostic PRGs upon the threshold of *P* < 0.2 to prevent omissions. Additionally, the R software “glmnet” function was utilized for LASSO analysis to construct a risk score model upon univariate regression. Later, our constructed model was adopted to determine the risk score for every ESCC case, and the median was adopted to classify cases as high- or low-risk groups. A forest was used to show the *P*-value, HR and 95% CI of each gene through the “forestplot” R package. Thereafter, the R software “stats” package was applied in PCA and tSNE.

### Development of the pyroptosis-related gene prognostic model

The survival probability was compared between the two subgroups via Kaplan-Meier analysis in TCGA. The predictive accuracy of each gene and the risk score were evaluated by performing survival probability analysis. Considering the clinical characteristics, a predicted nomogram was developed to predict the 1-, 3-, and 5-year overall survival. In addition, we verified whether our constructed risk model could predict prognosis based on the GSE53625 dataset.

### Functional annotation and changes in eight genes incorporated into the model

This work utilized the cBioPortal (https://www.cbioportal.org) dataset containing genome information of 104 cancer types for examining genetic variations of those genes incorporated into the model. GeneMANIA (http://www.genemania.org) provides genetic data, gene list information, functional annotation of key genes, and algorithms with great prediction ability. Hence, we adopted GeneMANIA for analyzing genes related to gene models and enrichment activities.

### Associations between genes/risk score and clinicopathological factors/immune cells/immune pathways

This study utilized the R package “beeswarm” function for evaluating the associations between genes/risk score and clinicopathological factors. Notably, the tumor immune microenvironment (TIME) represents an essential factor for antitumor immunity in the tumor. ssGSEA was conducted using the R package “gsva” function for calculating tumor-infiltrating immune cells (TIICs) scores and evaluating activities of immune pathways.

### Quantitative real-time PCR (qRT-PCR)

TRIzol reagent (Invitrogen, CA, USA) was used for extracting total RNA, which was later prepared into cDNA using the Prime Script RT Master Mix (TaKaRa, China). Thereafter, qRT-PCR was performed using SYBR Premix Ex Taq II (TaKaRa, China) for detecting target mRNA expression, while Bio-Rad CFX Manager software (Bio-Rad, USA) was used for data analysis, using GAPDH as the endogenous reference. Primer preparation was performed by Tsingke Biotechnology Co., Ltd. (China) 2^−ΔCt^ approach was employed for assessing mRNA expression. The sequences of primers used in this study are listed in Table 2.

**Table 2:** Primers of genes.

C2	F: GGGGACAAGGTCCGCTATC
	R: GAAGTCATAAGAGTAGGGTTGGC
CD14	F: ACGCCAGAACCTTGTGAGC
	R: GCATGGATCTCCACCTCTACTG
C1QB	F: ATGGGGCAGCATCCCAGTA
	R: CTCCCTTCTCTCCGAACTCAC
B2M	F: GAGGCTATCCAGCGTACTCCA
	R: CGGCAGGCATACTCATCTTTT
FCER1G	F: AGCAGTGGTCTTGCTCTTACT
	R: TGCCTTTCGCACTTGGATCTT
FCGR3A	F: CCTCCTGTCTAGTCGGTTTGG
	R: TCGAGCACCCTGTACCATTGA
RTP4	F: ACATGGACGCTGAAGTTGGAT
	R: TACGTGTGGCACAGAATCTGC
SLC7A7	F: CCCAAGGGTGTGCTCATATACA
	R: CCAGTTCCGCATAACAAAGGG

### Statistical analysis

The statistical analysis was carried out using R version 4.0.5, GraphPad Prism 9.0.0, and Perl version 5.28. Excel Office 2019 was used for GEO and TCGA databases. In univariate regression, prognostic PRGs were selected at *P* < 0.2. The significance level was set at *P* < 0.05 for the rest of analysis.

## RESULTS

### DEGs identification

**Figure 1:**
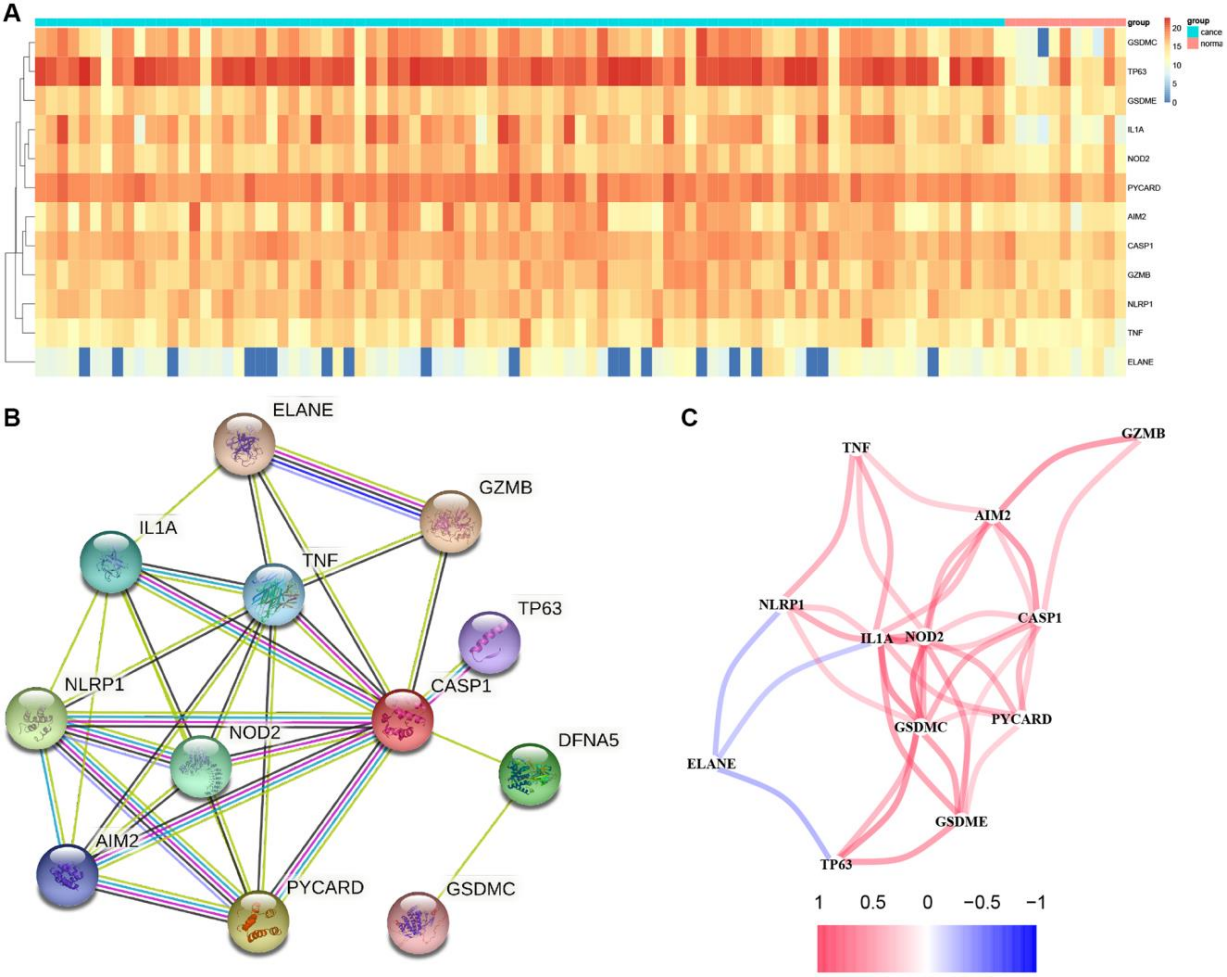
**Results of differential gene analysis.** (**A**) Heatmap of differentially expressed pyroptosis-related genes. The vertical axis refers to genes; the horizontal axis refers to differences in the gene expression between tissues, the red denotes high expression, and the blue denotes low expression. (**B**) PPI network showing the interactions of differentially expressed pyroptosis-related genes. (**C**) Correlation of the differentially expressed pyroptosis-related genes (Red line: Positive correlation; Blue line: Negative correlation. The depth of the colors reflects the strength of the relevance).

In total, 12 PRGs were selected through TCGA database analysis (Figure 1A). There were several of these genes upregulated in tumors compared to healthy samples, including Gasdermin C (GSDMC), Tumor Protein P63 (TP63), Nucleotide Binding Oligomerization Domain Containing 2 (NOD2), GSDME, Interleukin 1 Alpha (IL1A), PYD And CARD Domain Containing (PYCARD), Absent in Melanoma 2 (AIM2), Caspase 1 (CASP1), Granzyme B (GZMB), Tumor Necrosis Factor (TNF) and NLR Family Pyrin Domain Containing 1 (NLRP1), whereas Elastase, Neutrophil Expressed (ELANE) were downregulated. Using STRING database, we constructed a PPI network that CASP1, TNF, IL1A, NLRP1, AIM2 NOD2, and PYCARD as critical genes interacting with additional genes (Figure 1B). Figure 1C demonstrates a correlation network that contains 12 PRGs.

### Tumor classification according to differentially expressed PRGs (DEPRGs)

**Figure 2:**
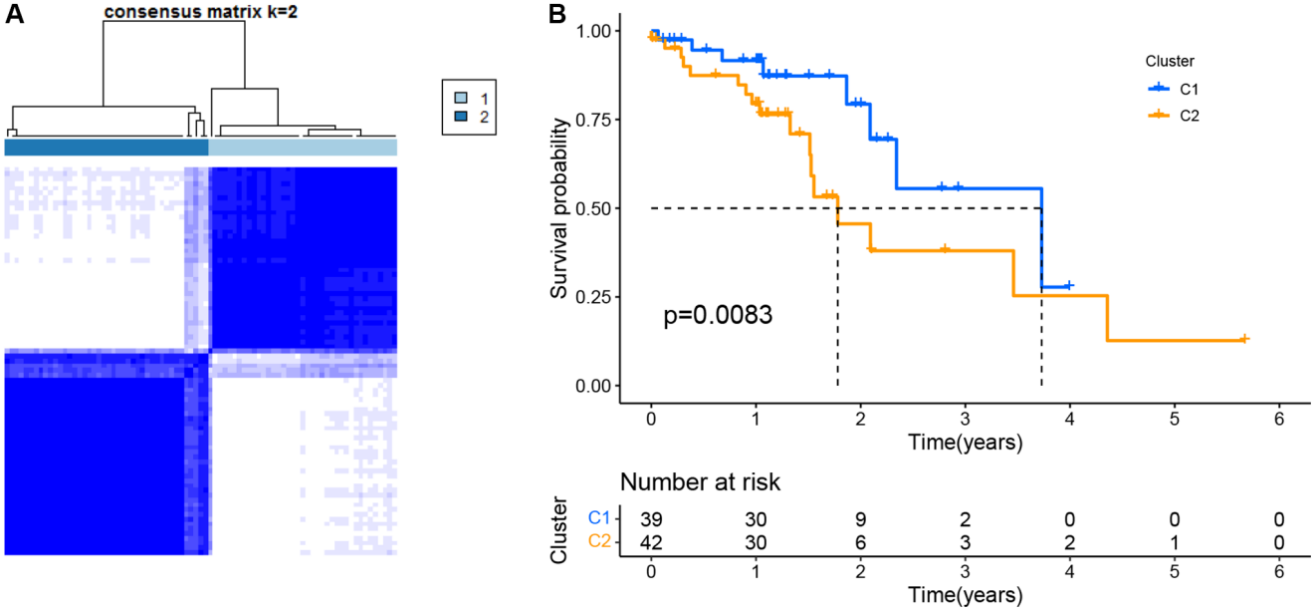
**Tumor classification as per the pyroptosis-related DEGs.** (**A**) ESCC patients were grouped into two clusters based on the consensus clustering matrix (k = 2). (**B**) Kaplan–Meier OS curves for the two clusters.

This work conducted consensus clustering analysis for analyzing the relations of 12 PRGs with ESCC subtypes based on TCGA-derived cases, and cases with < 30-day follow-up time were eliminated. With an increase in the clustering variable (k = 2–10), there were the greatest and smallest intragroup and intergroup connections, respectively, found at k = 2, which indicated that ESCC cases were classified as two clusters based on 12 DEGs (Figure 2A). Moreover, we compared the overall survival (OS) of both clusters, which exhibited significant differences (Figure 2B).

### Risk score model

**Figure 3:**
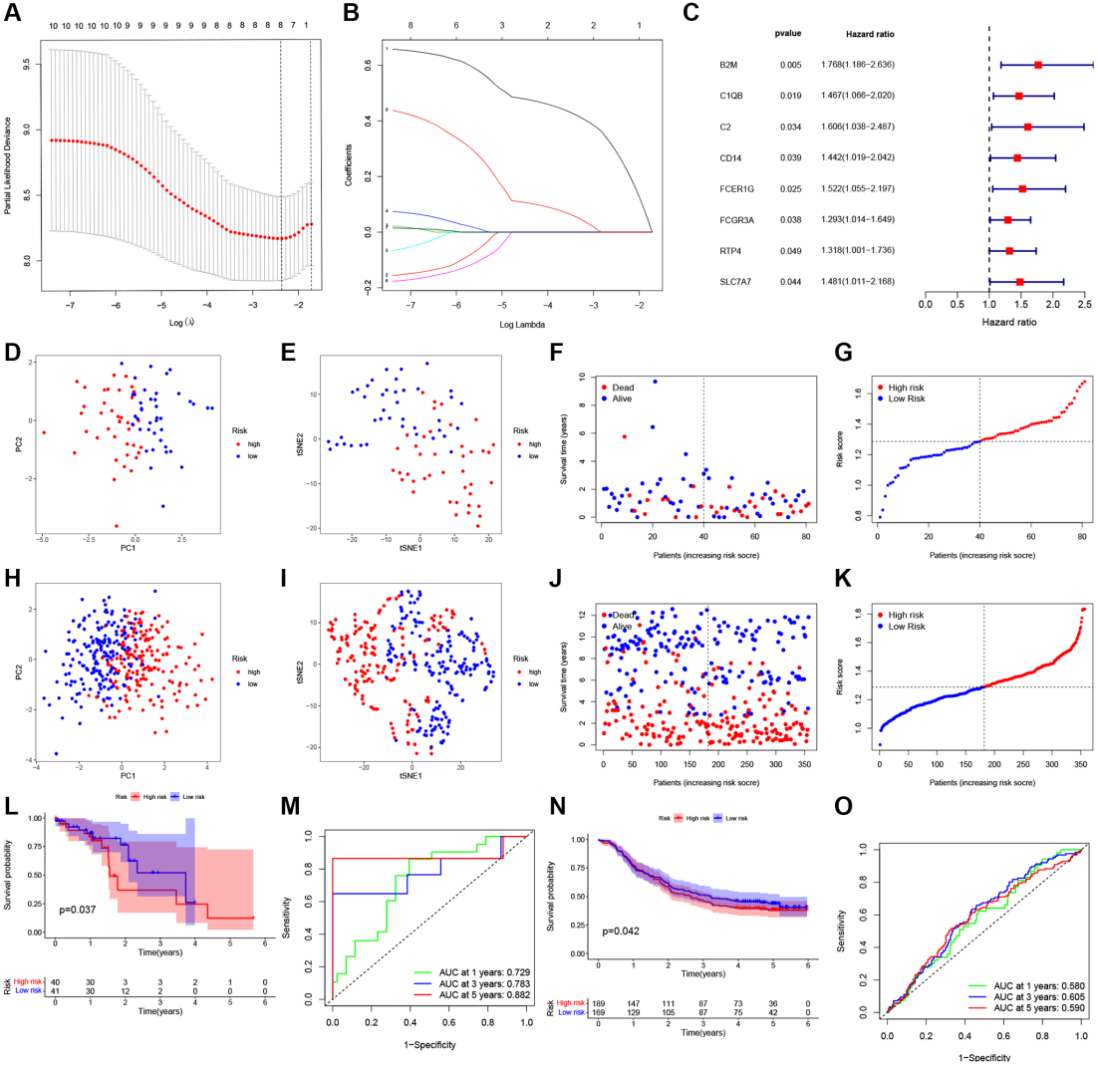
**Construction of a prognostic ESCC model.** (**A**) Distribution of LASSO coefficients for eight genes. Two vertical lines represent lambda. min and lambda. Lse. (**B**) Coefficients for eight genes analyzed by LASSO. (**C**) The hazard ratio of univariate Cox analysis for pyroptosis-related DEGs. (**D**) PCA plot for ESCC based on the risk score in TCGA. (**E**) tSNE plot for ESCC based on the risk score in TCGA. (**F**, **G**) Distribution of risk score, survival status in TCGA. (**H**) PCA plot for ESCC based on the risk score in GEO. (**I**) tSNE plot for ESCC based on the risk score in GEO. (**J**, **K**) Distribution of risk score, survival status in GEO. (**L**) Survival analysis to verify the prognostic model in TCGA. (**M**) Time-dependent ROC curves for ESCC in TCGA. (**N**) Survival analysis to verify the prognostic model in GEO. (**O**) Time-dependent ROC curves for ESCC in GEO.

For the present study, we used LASSO Cox regression to develop a risk model based on those eight PRGs (Figure 3A, 3B), and calculated risk scores for each case as follows, risk score = β1 × ExpmRNA1 + β2 × ExpmRNA2 + … + βn × ExpmRNAn. Furthermore, this work conducted univariate Cox regression by incorporating those chosen DEPRGs based on 275 ESCC cases. At *p* = 0.2, there eight genes were discovered (Figure 3C). By using principal component analysis (PCA) and T-Distribution Stochastic Neighbour Embedding (tSNE), TCGA and GEO cases with diverse risks were grouped into 2 clusters (Figure 3D, 3E, 3H, 3I). With the increase in risk score value, the mortality risk elevated accordingly, while survival time was shortened (Figure 3F, 3G, 3J, 3K). Moreover, based on our validation dataset, our constructed PRGs-based risk model performed well in predicting the patient prognosis (Figure 3L, 3N). In TCGA, the AUC values of 1-, 3-, and 5-year ROC curves were 0.729, 0.783, 0.882, respectively (Figure 3M), whereas those in GEO were 0.580, 0.605, 0.590, respectively (Figure 3O).

### Establishment of the PRG-based prognosis gene model

**Figure 4:**
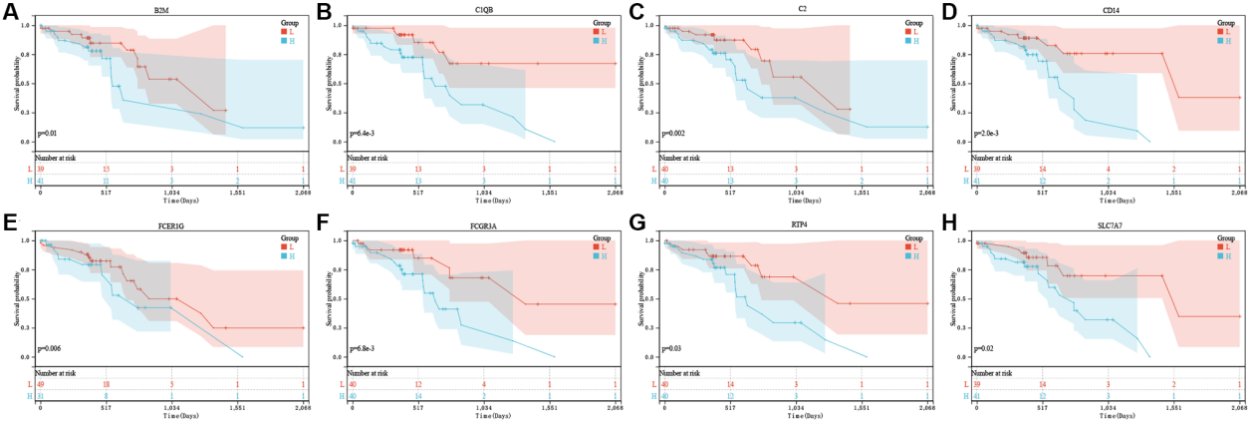
**The prognostic value of eight pyroptosis-related genes in ESCC.** In TCGA, the overall survival curve of B2M (**A**), C1QB (**B**), C2 (**C**), CD14 (**D**), FCER1G (**E**), FCGR3A (**F**), RTP4 (**G**), and SLC7A7 (**H**) in ESCC patients in the high-/low-expression group. PRG pyroptosis-related gene. Adjusted *P*-value < 0.05 is considered significant.

For constructing the prognosis gene model, this work used univariate Cox regression to select prognostic PRGs. Consequently, we detected eight prognostic PRGs altogether. Figure 4 represents K-M survival curves, indicating the dismal survival of ESCC. Cases showing upregulation of B2M (Figure 4A, *P* = 0.01), C1QB (Figure 4B, *P* = 6.4 × 10^−3^), C2 (Figure 4C, *P* = 0.002), CD14 (Figure 4D, *P* = 2.0 × 10^−3^), FCER1G (Figure 4E, *P* = 0.006), FCGR3A (Figure 4F, *P* = 6.8 × 10^−3^), RTP4 (Figure 4G, *P* = 0.03), SLC7A7 (Figure 4H, *P* = 0.02) are also represented.

### Univariate as well as multivariate Cox regression for risk score

**Figure 5:**
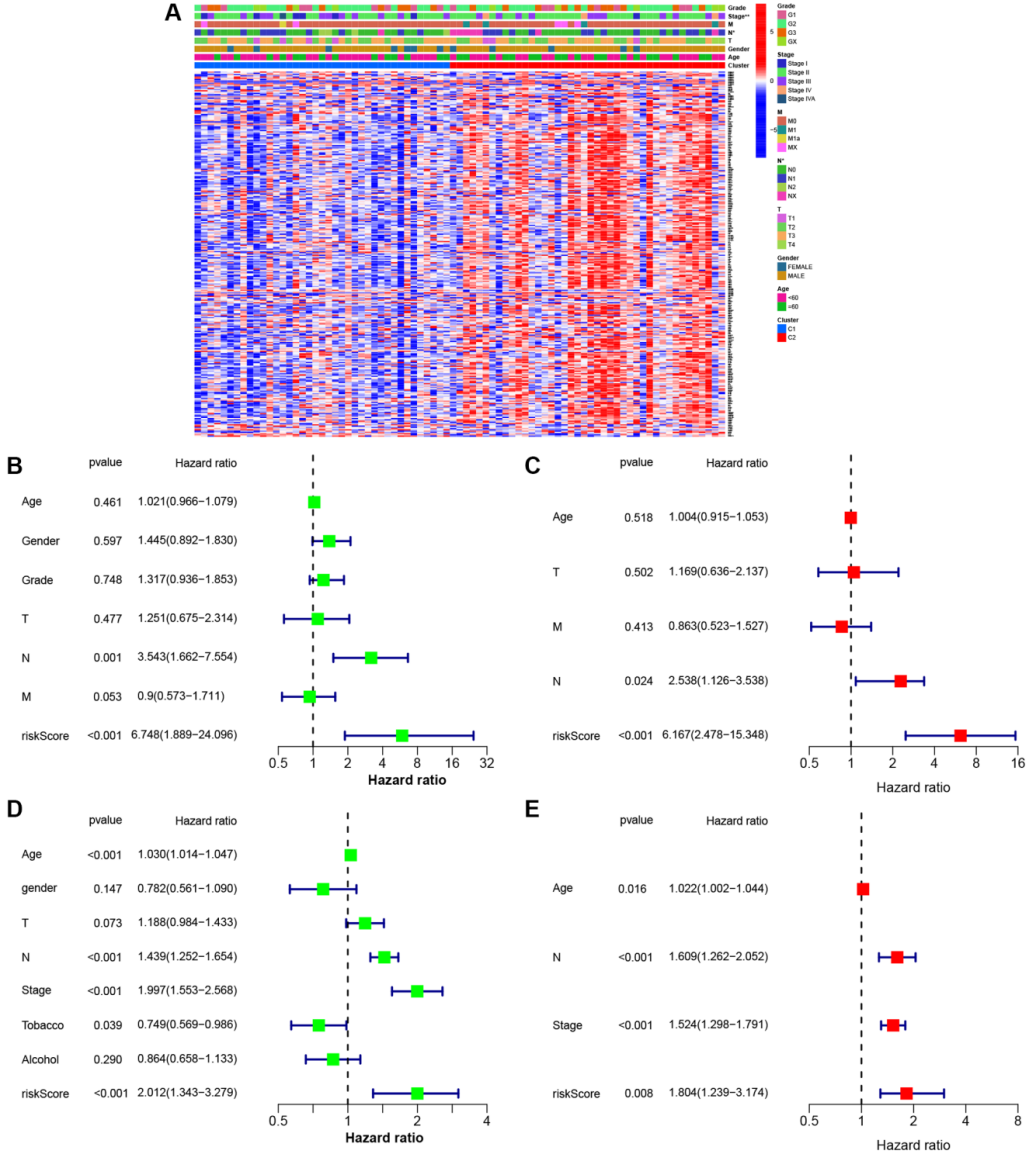
**Univariate and multivariate Cox regression analyses for the risk score.** (**A**) Heatmap (blue: low expression; red: high expression) for the connections between clinicopathological features and the risk groups (^*^P < 0.05). (**B**) Univariable Cox regression analysis for the risk score in TCGA. (**C**) Multivariable Cox regression analysis for the risk score in TCGA. (**D**) Univariable Cox regression analysis for the risk score in GEO. (**E**) Multivariable Cox regression analysis for the risk score in GEO.

Figure 5A represents eight gene expressions of ESCC cases and associated clinical data. Meanwhile, the heatmap described 12 diverse clinicopathological feature distributions depending on our risk model-determined risk scores of cases. Univariate and multivariate Cox regression was applied in assessing whether our as-constructed risk model independently predicted ESCC prognosis. According to Figure 5B–5E, our constructed risk model independently predicts ESCC prognosis.

### Functional annotation and construction of the novel prognosis signature according to ESCC molecular subtypes

**Figure 6:**
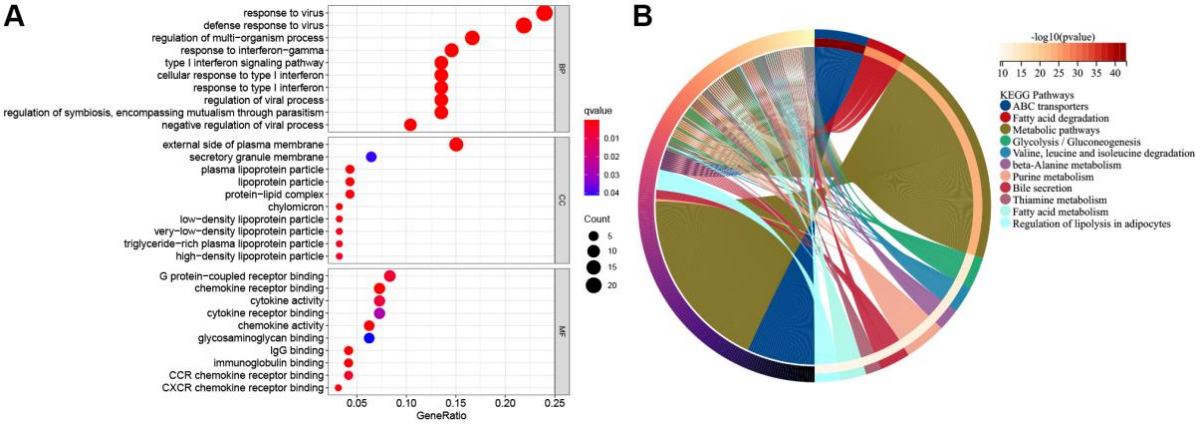
**The functional enrichment analysis of pyroptosis-related genes in ESCC.** (**A**) The enriched item in gene ontology analysis. The size of the circles represented the number of genes enriched. Abbreviations: BP: biological process; CC: cellular component; MF: molecular function; PRG: pyroptosis-related gene. The bigger bubble means more genes enriched, and the increasing depth of red denotes the differences were more obvious. *q*-value, the adjusted *P*-value. (**B**) The enriched item in Kyoto Encyclopedia of Genes and Genomes analysis.

For better understanding functions of candidate genes and pathways across diverse molecular subtypes, 293 DEGs were obtained from the TCGA cohort upon the thresholds of |log2FC|≥1 and FDR < 0.05. Thereafter, the selected DEGs were subjected to GO functional annotation and KEGG pathway enrichment. Figure 6A and 6B represents significantly enriched biological processes (BPs). Therefore, DEGs were associated with several biological and cellular activities, like G protein-coupled receptor binding, the external side of the plasma membrane, response to the virus, ABC transporters, fatty acid degradation, metabolic pathways and so on.

### Association between risk score and immune cells/immune function

**Figure 7:**
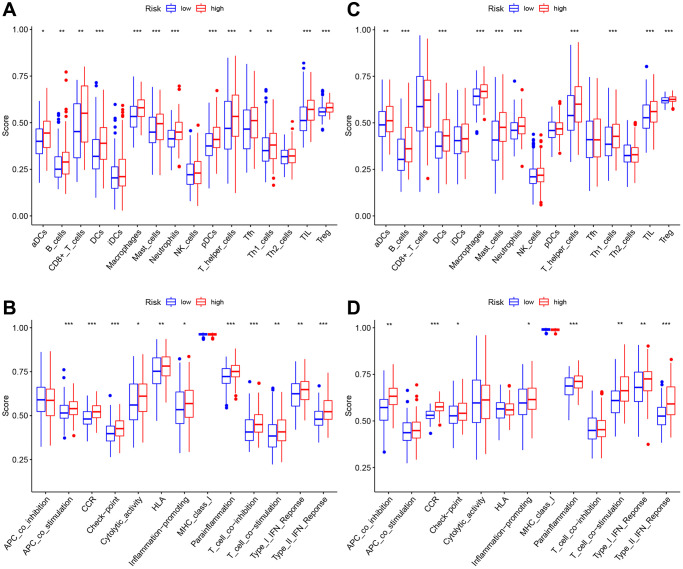
**The immune landscape of two pyroptosis-related molecular subtypes.** (**A**, **B**) Comparison of the enrichment scores of 16 types of immune cells and 13 immune-related functions between low- (blue box) and high-risk (red box) groups in the TCGA cohort, (**C**, **D**) Comparison of the enrichment scores of 16 types of immune cells and 13 immune-related functions between low- (blue box) and high-risk (red box) groups in the GEO cohort. Adjusted *P*-values were shown as ns (not significant); ^*^P < 0.05; ^**^P < 0.01; ^***^P < 0.001.

All TCGA-derived cases were classified as high- or low-risk groups based on the median risk score value. ssGSEA was conducted to compare enrichment scores for 16 TIICs and activities of 13 immune pathways between both groups. In TCGA-derived cases, high-risk patients had increased TIIC levels of activated dendritic cells (aDCs), B cells, CD8+T cells, DCs, macrophages, mast cells, neutrophils, plasmacytoid dendritic cells (pDCs), T-helper cells, Th1-cells, Tfh, Tregs and tumor-infiltrating lymphocytes (TILs) (Figure 7A). Figure 7B shows the enrichment of immune-related functions for high-risk patients. High-risk patients were clearly associated with increased APC co-stimulation, checkpoint, CCR, HLA, cytolytic activity, parainflammation, inflammation-promoting, T cell-co-stimulation, T cell co-inhibition, Type I and Type II IFN responses. In GEO-derived cases, high-risk patients had increased TIIC levels of aDCs, B cells, DCs, macrophages, mast cells, neutrophils, Th1-cells, T-helper cells, Tregs, and TILs (Figure 7C). Figure 7D shows the enrichment of immune-related functions for high-risk patents. Clearly, high-risk patients were associated with the increased APC co-inhibition, checkpoint, CCR, parainflammation, inflammation-promoting, T-cell-co-stimulation, Type I and Type II IFN responses.

### Eight genes expression at mRNA levels

**Figure 8:**
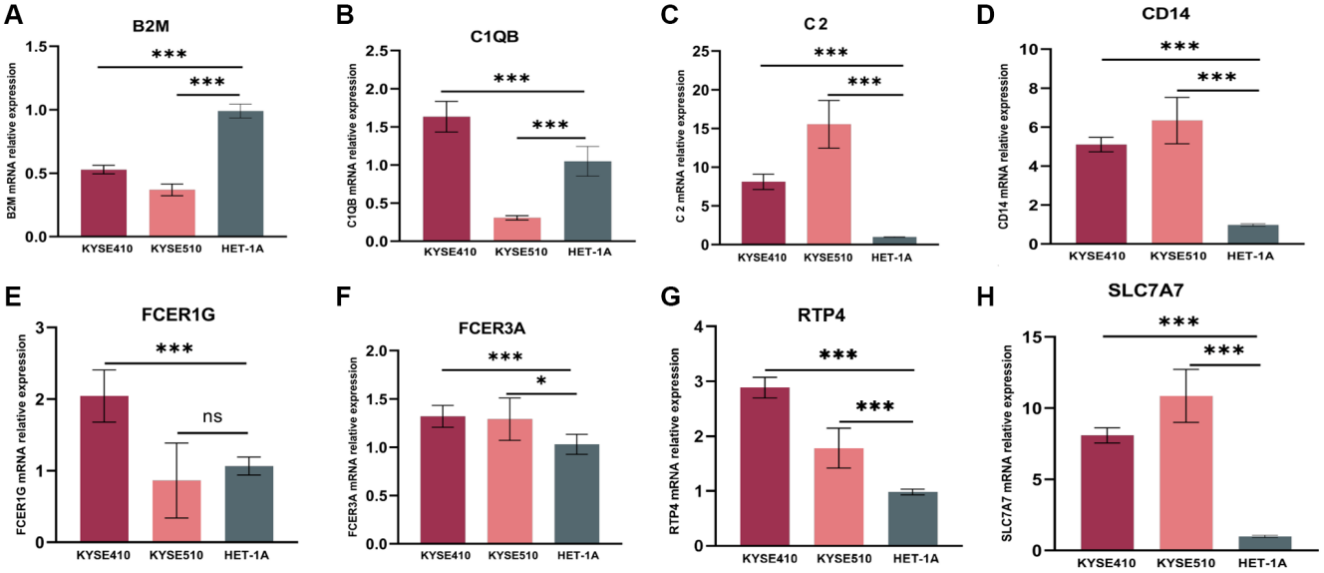
**mRNA relative expression of genes in the risk model by the method of qPCR.** (**A**) mRNA relative expression of B2M. (**B**) C1QB. (**C**) C2. (**D**) CD14. (**E**) FCER1G. (**F**) FCER3A. (**G**) RTP4. (**H**) SLC7A7. GAPDH expression was used as an internal control. qPCR, quantitative real-time polymerase chain reaction.

As confirmed by ESCC cells (KYSE410, KYSE510) and normal epithelium of esophagus (HET-1A) obtained from Hunan Fenghui Biotechnology Co., Ltd. (Changsha, China) through qPCR, C2, CD14, RTP4, FCER3A, and SLC7A7 were highly expressed in KYSE410 and KYSE510 than normal cells (Figure 8A–8H). The expression of B2M, C1QB, and FCER1G did not show a significant difference, which could be a result of individual variability requiring an increased sample size.

## DISCUSSION

The findings suggested that the PRG-based gene risk model might be adopted for predicting ESCC prognosis. Also, our as-constructed risk model was associated with clinicopathological characteristics, immune cells, as well as immune activities. Pyroptosis represents a novel type of programmed cell death that has been widely investigated in a number of disorders in recent years [14]. It may result from canonical caspase-1 inflammasomes or from caspase-4/5/11 activation by cytosolic lipopolysaccharide [15]. Pyroptosis may affect cancer growth, migration, and invasion [16]. The effects of GSDME and Gasdermin A3 (GSDMA3) on suppressing cancer proliferation by promoting cytotoxic responses of lymphocytes are reported [12]. After chemotherapy, the degradation of GSDME by caspase-3 can cause some cancer cells to scorch death [17]. PD-L1 can alter TNFα-mediated tumor cell apoptosis into pyroptosis, leading to tumor necrosis [18]. When ESCC is treated with DHA, certain dying cells show the typical pyroptosis morphologies, such as blowing huge bubbles from the cell membrane, accompanied by reduced expression of pyruvate kinase isoform M2 (PKM2), GSDME, and caspase-3/8 activation, together with GSDME-NT generation [19]. Cisplatin exposure increased ROS levels, which activated GSDME and caspase-3 and promoted ESCC cell pyroptosis [20]. PDT inhibited PKM2 expression, thereby activating caspase-3/8 and releasing N-GSDME while triggering ESCC pyroptosis [21]. BI2536 (PLK1 inhibitor) can enhance the DDP sensitivity of ESCC cells by promoting pyroptosis while suppressing the DNA damage repair pathway. A combination of BI2536 and DDP treatment led to ESCC cell pyroptosis via the caspase-3/GSDME pathway [22]. Metformin induces ESCC pyroptosis via miR-497/PELP1 pathway [23]. Disruption of circPUM1 resulted in pyroptosis of ESCC cell lines [24]. These studies suggested that many chemotherapies and targeted drugs work by causing ESCC cells to pyroptosis, and PRGs are possible biomarkers for ESCC. However, there is few article using PRGs for constructing the risk model of ESCC. In this work, the TCGA dataset was used to be the training set, whereas the GEO dataset to be the validation set for constructing an eight PRGs-based risk model based on univariable as well as LASSO Cox regression. Therefore, our prognostic model predicted prognosis well, which could be useful for clinical assessment and discovering novel therapeutic targets. Genes incorporated into our as-constructed risk model are recognized in cancer research. β2-microglobulin (B2M) mutation is found to be the immune escape genetic mechanism in anti-programmed cell death protein 1 (PD-1) treatment [25]. HLA class I antigen deletion caused by B2M mutation mostly occurs under the activated PDCD1 (PD-1)-positive T cell infiltration condition [26]. C1QB is the diagnostic and prognostic biomarker for skin cutaneous melanoma patients [27], IRF4 could promote melanoma cell growth via upregulating C1QB [28]. The combination of C2 and additional therapeutic mAbs, such as type II anti-CD20/CD22/CD38 samples, can overcome complement attack resistance in tumor cells [29]. Genetic variability in CD14 may play a role in developing gastric cancer precursor lesions over time and in gastric carcinogenesis [25]. PD-L1 CD14 monocytes are markedly associated with OS of different cancers after anti-PD-1 blockade treatments [30]. CD14+ monocytes from peripheral blood in renal cell carcinoma (RCC) cases show remarkable phenotypic changes, which are five times greater than the mean value found in normal subjects. CD14+ cells present around and inside the tumor might independently predict the patient prognosis [31]. Cancer cells with high CD14 expression show increased amounts of many inflammatory factors, resulting in greater tumor formation than cells with low CD14 expression. The inflammatory factors generated by the high CD14 expression bladder cancer cells recruit and polarize macrophages and monocytes to acquire immune-suppressive characteristics. Bladder cancer cells with high CD14 can mediate tumor-promoting inflammation while driving cancer cell growth for promoting tumor development [32]. The expression of FCER1G increases within many cancers. FCER1G expression was positively associated with tumor prognostic outcome, growth, and migration; also, FCER1G was closely related to tumor immunity and tumor microenvironment (TME) [33]. FCGR3A usually shows upregulated in pan-cancer [34]. In survival analysis, FCGR3A has been found to be a major risk factor for many tumors [35]. Besides, FCGR3A expression is associated with infiltrating degrees of certain immune cells [36], levels of DNA mismatch repair genes, and numerous immune checkpoint genes. Based on the drug sensitivity analysis, FCGR3A upregulation predicts the decreased IC50 values of many drugs [34]. RTP4 depends on the infiltrating degrees of immune cells. Furthermore, it showed a close association with genes that encode immune checkpoint components (PDCD1, LAG3, TIM-3) [37]. Glioma risk may be affected by SLC7A7 genetic variants [38]. Based on the above findings, we identified eight PRGs related to the tumor. Our risk model built using these PRGs was associated with the infiltrating degrees of immune cells (such as neutrophils and macrophages). Thus, our as-constructed risk model is associated with TME and could be used to predict ESCC prognosis. However, there are some limitations associated with it. Firstly, this risk model needs to be further validated with more data. Secondly, certain PRGs incorporated in this model should be validated with more samples, and their underlying mechanisms ESCC should be further explored.

In summary, this work suggested that PRGs were differentially expressed in healthy compared with ESCC samples. Furthermore, according to eight PRGs, our constructed risk score independently predicts the prognosis of ESCC. Therefore, our findings contribute to identifying early cases and offer candidate novel antitumor therapeutic targets.
